# Valorization of sesame seed coat waste: phenolic composition, antibacterial efficacy, and nanoemulsion encapsulation for food preservation

**DOI:** 10.3389/fnut.2024.1405708

**Published:** 2024-06-14

**Authors:** Salma Khazaal, Mahmoud I. Khalil, Tareq M. Osaili, Borhan Albiss, Anas A. Al-Nabulsi, Nicolas Louka, Nada El Darra

**Affiliations:** ^1^Department of Biological Sciences, Faculty of Science, Beirut Arab University, Beirut, Lebanon; ^2^Molecular Biology Unit, Department of Zoology, Faculty of Science, Alexandria University, Alexandria, Egypt; ^3^Department of Clinical Nutrition and Dietetics, College of Health Sciences, University of Sharjah, Sharjah, United Arab Emirates; ^4^Department of Nutrition and Food Technology, Faculty of Agriculture, Jordan University of Science and Technology, Irbid, Jordan; ^5^Nanomaterials Laboratory, Department of Applied Physics, Jordan University of Science and Technology, Irbid, Jordan; ^6^Centre d'Analyses et de Recherche, Unité de Recherche Technologies et Valorisation Agro-Alimentaire, Faculté des Sciences, Université Saint-Joseph de Beyrouth, Beirut, Lebanon; ^7^Department of Nutrition and Dietetics, Faculty of Health Sciences, Beirut Arab University, Beirut, Lebanon

**Keywords:** sesame seed coat, valorization, polyphenols, antibacterial activity, nanoemulsion

## Abstract

The study highlighted the potential of sesame seed coat (SSC), typically discarded during sesame paste processing, as a valuable resource for valorization through extracting bioactive compounds. It examined the phenolic composition and antioxidant activity of SSC, and evaluated its antibacterial properties against foodborne pathogens such as *Listeria monocytogenes, Escherichia coli* O157:H7, and *Salmonella* Typhimurium. Additionally, SSC underwent nanoemulsion coating, analyzed using dynamic light scattering and scanning electron microscopy, to enhance its application as a natural preservative. The research specifically focused on incorporating SSC nanoemulsion into milk to determine its effectiveness as a preservative. SSC demonstrated considerable antioxidant activity and phenolic content, with catechin identified as the predominant polyphenol. GC-MS analysis revealed seven major compounds, led by oleic acid. Notably, SSC effectively inhibited *L. monocytogenes* in broth at 100 mg/ml. The application of SSC and its nanoemulsion resulted in changes to bacterial morphology and a significant reduction in bacterial counts in milk, highlighting its potential as an effective natural antibacterial agent. The findings of this study highlight the potential use of SSC as a valuable by-product in the food industry, with significant implications for food preservation.

## 1 Introduction

Sesame (*Sesamum indicum* L.), a member of the *Pedaliaceae* family, is a vital oilseed crop worldwide (1–3). Nutritionally, several studies have highlighted the chemical composition of sesame seeds: oil (44–58%), protein (18–25%), carbohydrate (13.5%), and ash (5%) (4–7). In 2022, the world production of sesame seeds was almost 7 million tons (8). In the Eastern Mediterranean regions, the seed undergoes industrial processing to produce sesame oil, sesame paste (also known as tahini), and halva (sweetened tahini) (9). During the processing of tahini or halva, sesame seeds are dehulled and a by-product is produced in large quantities, which is the sesame seed coat (SSC) (10). The SSC represents nearly 12% of the sesame seed (11). SSC has been recognized as a crucial industrial waste, mainly because of its significant biological properties associated with its composition. As indicated by the reports, this waste is a valuable source of dietary fibers, oil, and polyphenols, with quantities of 42, 12.2, and 0.6 g/100 g seed coat, respectively (10, 12). However, in several countries that process sesame, it is generally discarded or used for animal feed (13, 14). In recent times, reducing industrial waste has become a matter of great concern due to its adverse impact on the environment, health, and economy. Consequently, researchers are exploring ways to valorize this waste as a primary material to create valuable products, contributing to a sustainable circular bioeconomy with zero waste. Therefore, the SSC can be given an added value by extracting its vital bioactive compounds, particularly polyphenols.

Phenolic compounds include simple molecules such as gallic acid, caffeic acid, vanillin, and others, along with complex polyphenols such as flavonoids (15). Studies have verified that most phenolic compounds possess antimicrobial, antioxidant and anticarcinogenic activities (16–18). Several techniques have been developed to extract polyphenols from plants. The traditional water bath (WB) method is commonly used due to its ease of use, despite its limitations such as excessive energy usage (19). However, polyphenols tend to be unstable once extracted from plant materials. They are susceptible to degradation and exhibit a strong sensitivity to a range of environmental factors such as pH, temperature, exposure to oxygen, light, and moisture. This sensitivity leads to a reduction in their nutritional and functional qualities when stored for extended periods (20). This highlights the need for innovative approaches to preserve the bioactive components. In recent years, the exploration of biologically active formulations at the nanoscale has gained attention to overcome these challenges. Nanoemulsions, with their small particle size (50 to 1,000 nm), enhance interactions with biological membranes, ensuring efficient permeation. The customizable properties of these compounds, such as their high kinetic stability and low turbidity, make them well-suited for a wide range of applications. By addressing the sensitivity and stability issues commonly associated with biologically active compounds, they provide an effective solution for many needs (21–23).

While synthetic preservatives have traditionally been effective in combating food spoilage, concerns about their potential health risks have led to restrictions or bans in certain countries. Safer alternatives derived from natural sources have been developed to address these concerns (24). In this context, the objective of this study was to evaluate the antibacterial activity of the SSC extract encapsulated in a nanoemulsion against *Listeria monocytogenes* and *Escherichia coli* O157:H7. To achieve this, a model medium was adopted in the form of previously sterilized milk deliberately inoculated with these two microorganisms.

The main objective of this work was to valorize sesame by-product (SSC) obtained during food processing, which is typically discarded. We have accomplished this by extracting the bioactive compounds from the SSC and encapsulating them. This approach helps preserve the bioactive compounds present in the SSC, enabling them to be incorporated into food products as a natural preservative.

## 2 Materials and methods

### 2.1 Materials

The sesame seed coats (SSC) were obtained from a Lebanese industry that processes sesame paste (tahini). It should be noted that the sesame seeds are imported from Gadarif State-Sudan. Specifically, the SSC used in our study were obtained by mixing and processing together the coats from three different lots. The following chemicals and reagents were obtained from Sigma Aldrich (Steinheim, Germany): methanol (99.8%), Folin–Ciocalteu reagent, sodium carbonate (Na_2_CO_3_), gallic acid (3,4,5-trihydroxybenzoic acid), DPPH (2,2-diphenyl-picrylhydrazyl), Trolox (6-hydroxy-2,5,7,8-tetramethylchromane-2-carboxylic acid), and dimethylsulfoxide (DMSO; HPLC grade), chitosan (low molecular weight), glacial acetic acid, tween80 along with all HPLC standards (gallic acid, catechin, caffeic acid, p-coumaric acid, and quercetin). Polyethylene glycol was purchased from MERCK-Schuchardt.

Brain Heart Infusion (BHI), Tryptone Soya Broth (TSB), Mueller-Hinton Broth (MHB), Tryptone Soya Agar (TSA), Listeria Selective Agar Base, Sorbitol MacConkey Agar, Modified Listeria Selective Supplement, Cefixime Tellurite Selective Supplement, and peptone water were purchased from Oxoid (Basingstoke, UK). All media were prepared according to the manufacturer's instructions, except peptone water, which was prepared by dissolving 1 g in 1 liter of distilled water to obtain a 0.1% solution.

### 2.2 Water bath extraction method

WB extraction was carried out using a digital water bath (JSR JSWB-22T, Gongju City, Korea). Before extraction, the SSCs were sieved to obtain a particle size of < 500 μm and stored in the dark at room temperature. Ten g of SSCs were placed in 100 ml of 70% methanol solution (ratio 1/10), covered with aluminum foil, at 74°C for 17.5 min in the water bath, based on a previous study (25). The extracts were filtered through Whatman No. 1 filter paper and centrifuged for 10 min at 4,500 g/min. The supernatant was filtered through a 0.22 μm polyvinylidene fluoride (PVDF) syringe filter and stored at −80°C.

### 2.3 Sesame seed coats processing

SSC extracts were concentrated using a rotary evaporator and then lyophilized (Edwards freeze-dryer, UK) for 48 h. Freeze-dried samples were converted into powder with the help of a pestle and mortar (26). For all subsequent analyses, SSC was utilized in its lyophilized form. In order to calculate the yield of the lyophilized extract, the weight of the extract after lyophilization was divided by the initial weight of the plant material.

### 2.4 Determination of the total phenolic content

TPC was determined according to the Folin–Ciocalteu method described previously in the literature (27). A volume of 200 μl of the SSC extract (100, 50, 25, and 12.5 mg/ml) and 1,000 μl of the Folin-Ciocalteu reagent (diluted 1/10 v/v) were added to 800 μl of Na_2_CO_3_ 7.5% (w/v). The mixture was then incubated for 10 min at 60°C, followed by an additional 10 min at 4°C. The absorbance was measured at 750 nm using a UV-vis spectrophotometer (GENESYS 10 UV, Thermo Electron Corporation, Waltham, MA, USA). A calibration curve using gallic acid as the standard compound was employed for quantification. The TPC was expressed as milligrams of gallic acid equivalents (GAE) per gram of dry matter (mg GAE/g of DM).

### 2.5 DiPhenyl-2-PicrylHydrazyl free radical scavenging activity

The free radical scavenging activity was evaluated by measuring the ability of phenolic compounds to reduce the DPPH radical (28). Briefly, 1.45 ml of DPPH (0.06 mM) was added to 50 μl of SSC extract (100, 50, 25, and 12.5 mg/ml) or Trolox (positive control) or methanol (negative control). After 30 min of incubation at room temperature in the dark, the absorbance was measured at 515 nm. Pure methanol was used as the blank, and a calibration curve was made using Trolox as the standard. The antioxidant activity of the extracts was calculated according to the following formula:


$$\begin{array}{l} Antioxidant\;activity\;percentage\;\left(\%\right)=\\ \frac{Abs\;\left(negative\;control\right)\;-\;Abs\;\left(sample\right)}{Abs\;\left(negative\;control\right)}\;\times \;100 \end{array}$$

### 2.6 High-performance liquid chromatography with diode-array detection

Phenolic compounds in the extracts obtained from SSC were analyzed using HPLC-DAD. The HPLC system used for this analysis was an Agilent 1,100 series system equipped with an autosampler, a Zorbax column oven, and a diode array detector. To separate phenolic compounds, a C18 column (250 × 4.6 mm; 5 μm) was used. The following standards were used for identification and quantification: gallic acid, catechin, caffeic acid, p-coumaric acid, and quercetin. The mobile phase consisted of acetonitrile (A) and water/phosphoric acid pH 2 (B) of HPLC grade. The flow rate was set at 1 ml/min and the gradient program was as follows: 95% B to 65% B in 20 min, 65% B to 60% B in 10 min, 60% B to 50% B in 10 min, 50% B to 30% B in 12 min, and 30% B to 95% B in 8 min. The injection volume was 50 μl. The phenolic compounds were identified by comparing the retention times of the observed peaks with those of the standard compounds. To determine the concentration of these phenolic compounds, standard curves for each specific compound were established by employing various concentrations of the corresponding standards (Supplementary Figure 1).

### 2.7 Gas chromatography coupled with mass spectrometry for SSC extract

The chemical profile of the SSC was assessed following the methodology described by Qadir et al. (29). A gas chromatograph (GC) (SHIMADZU QP2010, Japan) coupled with mass spectrometry (MS) featuring a fused-silica capillary column was employed, with a mobile phase carrier gas (helium) set at a flow rate of 1.69 ml/min. The oven temperature was set at 50°C for 1 min followed by an increase of 3°C/min to 280°C. The total running time was 27 min.

### 2.8 Preparation of bacterial cultures

Bacterial cultures used in this study included *Listeria monocytogenes* (ATCC 7644), *Salmonella* Typhimurium (02:8423 strain), and *Escherichia coli* O157:H7 (02:0627 strain). These cultures were individually preserved in BHI broth containing 20% glycerol (v/v) at −20°C. For each working culture, the stock bacteria were streaked on TSA and then incubated at 37°C for 24 h to obtain a single colony. Subsequently, subculturing was performed on TSB, except for *L. monocytogenes*, where the TSB was supplemented with 0.6% yeast and incubated at 37°C for 24 h. The final concentration of bacterial cells was adjusted to 10^6^ CFU/ml.

### 2.9 Determination of antibacterial activity of SSC extracts

The antibacterial activity against each of the three bacterial strains was determined using the microdilution method. The highest concentration of lyophilized extract was obtained by dissolving 100 mg in 1 ml of 0.5% DMSO and then serially diluted twice to obtain concentrations of 50 mg/ml and 25 mg/ml. In a 96-well microtiter plate, 100 μl of each bacterial culture was mixed with 100 μl of extract. A 0.5% DMSO solution mixed with each bacterial strain served as a positive control, while DMSO containing the extracts was used as a negative control. The sealed microplate was then incubated for 24 h at 37°C. One hundred μl aliquots of each sample were taken and decimal dilutions with 0.1% peptone water were prepared. To determine the minimal inhibitory concentration (MIC) of each extract, the lowest concentration at which no visible growth is observed compared to the negative and positive controls should be identified. However, because of the dark color of the extract and the difficulty of detecting growth, the minimal bactericidal concentration (MBC), which is the lowest concentration of extract that kills the entire bacterial population, was also determined.

For this, 100 μl from each of the appropriate dilutions was spread on the surface of TSA and incubated for 24 h at 37°C (30).

### 2.10 Formation of SSC nanoemulsion

An aqueous solution was first prepared by dissolving 0.5 g of chitosan in 1,000 ml of 2% glacial acetic acid by stirring at 750 rpm for 30 min. Then, this solution was centrifuged at 4,500 g for 10 min (31). To 25 ml of the upper solution, 0.125 g of polyethylene glycol was added and stirred for 20 min. To prepare the nanoemulsion, the SSC extract was first mixed with 0.1% of Tween 80. Then, this phase was gradually added dropwise to the chitosan solution at a rate of 0.1 ml/min while on a magnetic mixer. The solution was stirred for 30 min to ensure the formation of the SSC-chitosan nanoemulsion (NE-SSC). The control solution (NE) was obtained by the same procedure but without the SSC for comparison.

### 2.11 Characterization of nanoemulsion

#### 2.11.1 Dynamic light scattering

To determine the average particle size and surface charge of NE-SSC and NE, zeta potential analysis was conducted using a Malvern Zetasizer ZS instrument (Instruments Limited, ZEN3600, UK). Fresh disposable polystyrene cuvettes were used for each sample for dynamic light scattering (DLS), and data was analyzed using the Malvern Zetasizer v7.11 software (Malvern Corp., Malvern, UK). The software used appropriate refractive indices for the bulk material and the solvent, modeling each particle as a sphere. To avoid multiple scattering, the samples were diluted in deionized water in a 1:20 (v/v) ratio (26).

#### 2.11.2 Scanning electron microscopy

The morphology and shape of NE-SSC (nanoemulsion) and NE (control) were examined using Thermo Fisher's SEM (FEI Quanta 450 FEG SEM; USA). Imaging was performed in high vacuum at an acceleration voltage of 30 kV. The samples were mounted on aluminum stubs by double-sided sticky disks of conductive carbon and then gold coated by sputter coater (Quorum Q150R ES, Quorum Technologies Ltd. Ashford. Kent. England).

### 2.12 Atomic force microscopy

#### 2.12.1 Preparation of mica sheets

To prepare bacterial cells for imaging, we utilized mica sheets (Ted Pella Inc., Redding, CA, USA) as the substrate for immobilizing the samples. The procedural steps for preparing the mica slides followed the methodology outlined by Allison et al. (32). Initially, the mica slides were trimmed to a suitable size compatible with the microscope (approximately 10 × 22 mm). Subsequently, the outer layers of the slides were removed on both sides using tape. The next step involved coating the slides with gelatin by quickly immersing and withdrawing them in a warm gelatin solution (0.5 g/100 ml of distilled water at 60–70°C). After coating, the slides were left to air dry overnight on a paper towel while supported on an edge beneath a biosafety cabinet to prevent any risk of contamination.

#### 2.12.2 Preparation and mounting of bacterial cells

To obtain images of bacterial cells, gelatin-coated mica was used to mount the cells. Bacterial culture was prepared for both the control and treated groups. For the control group, sterilized distilled water was used to introduce bacteria (OD: 0.5–1.0 at 600 nm). The resulting mixtures were incubated for 24 h at 37°C, then centrifuged (4,500 rpm for 10 min) and washed with sterilized distilled water. The pellets were mixed with 50 μl of distilled water and vortexed. Then 20 μl of the solution was applied to a mica slide, allowed to dry, and washed with distilled water. The slides were then prepared for imaging. For the SSC-treated bacteria, 1 ml of each bacterial culture in MHB (OD: 0.08–0.13 at 600 nm) was combined with 1 ml of SSC at a concentration of 100 mg/ml or 1 ml of NE-SSC and subjected to the same procedure. To image the bacterial cells, AFM non-contact mode, n-type silicon tip (NSC15/Al BS, MikroMasch, Estonia), nominal spring constant of 40 N/m, resonance frequency of 356 kHz, and a scanning rate of 0.3–0.7 Hz were employed.

### 2.13 Antibacterial activity in milk

#### 2.13.1 Bacterial cultures

The bacterial cultures (*L. monocytogenes* and *E. coli* O157:H7) were prepared as mentioned previously in Section 2.8, except that after incubation in TSB, the bacterial cultures were centrifuged and washed with 0.1% peptone water 3 times. To make a comparison between Gram-positive and Gram-negative bacteria, one of each type was selected. As a result, *S*. Typhimurium was not included in this part of the study. A sterilized milk, purchased from the local market, was inoculated with each bacterial strain to give a concentration of 10^4^ CFU/ml.

#### 2.13.2 Antibacterial activity of SSC in milk

The SSC concentration at the highest concentration (100 mg/ml) was selected to evaluate its effectiveness against *L. monocytogenes* and *E. coli* O157:H7 in sterilized milk. All steps were performed under aseptic conditions. In a 96-well microtiter plate, 100 μl of each inoculated milk was mixed with 100 μl of SSC. The sealed microplate was then placed in incubation for different durations (0, 1, 3, and 7 days) at two different temperatures, specifically 4 and 10°C. The bacterial survival count, expressed in log CFU/ml, was achieved in an appropriate selective medium, Listeria selective agar base for the *L. monocytogenes* and sorbitol MacConkey agar for *E. coli* O157:H7, overlaid with TSA to recover the injured cell (33). Plates were incubated for 24 h at 37°C.

#### 2.13.3 Antibacterial activity of NE-SSC in milk

The antibacterial activity of NE-SSC was determined in sterilized milk. In a 96-well microtiter plate, NE-SSC and NE at different concentrations (0%, 3%, 7%, and 10%) were mixed with inoculated milk with a fixed amount of sterilized milk. The sealed microplate was then placed for incubation at different durations (0, 1, 3, and 7 days) and at two different temperatures, specifically 4 and 10°C. At each time point (0, 1, 3, and 7 days) and temperature (4 and 10°C), 100 μL aliquots of each sample were taken and decimal dilutions with 0.1% peptone water were prepared. Following that, 100 μl from each of the appropriate dilutions were spread onto selective bacterial media surfaces (as mentioned previously) and incubated for 24 h at 37°C.

### 2.14 Statistical analysis

The experiments were carried out three times (*n* = 3), and the mean value ± standard error of mean (SEM) value was used to present the results. Both control and experimental groups were analyzed and *p*-values < 0.05 were considered statistically significant, indicating a confidence level >95%. Statistical analysis of the data involved independent *t-*test (unpaired 1-tailed) and Analysis of Variance (ANOVA) followed by Duncan Multiple Range Test (DMRT) using IBM SPSS Statistics for Windows, Version 25.0 (Released 2017. IBM Corp., New York, USA).

## 3 Results and discussion

### 3.1 Extraction yield, total phenolic content and antioxidant activity of SSC extract

The extraction yield in this study was 10.62 ± 0.42 %, which is consistent with the study by Elleuch et al. (13) on extracting sesame seed coat using 70% methanol, which yielded almost 10.5%. The extraction yields in this study were greater than those reported by Chang et al. (34) (8.19%) for 100% methanol extraction. This suggests that increasing the water content of the solvent may lead to an increase in the yield (34).

**Figure 1:**
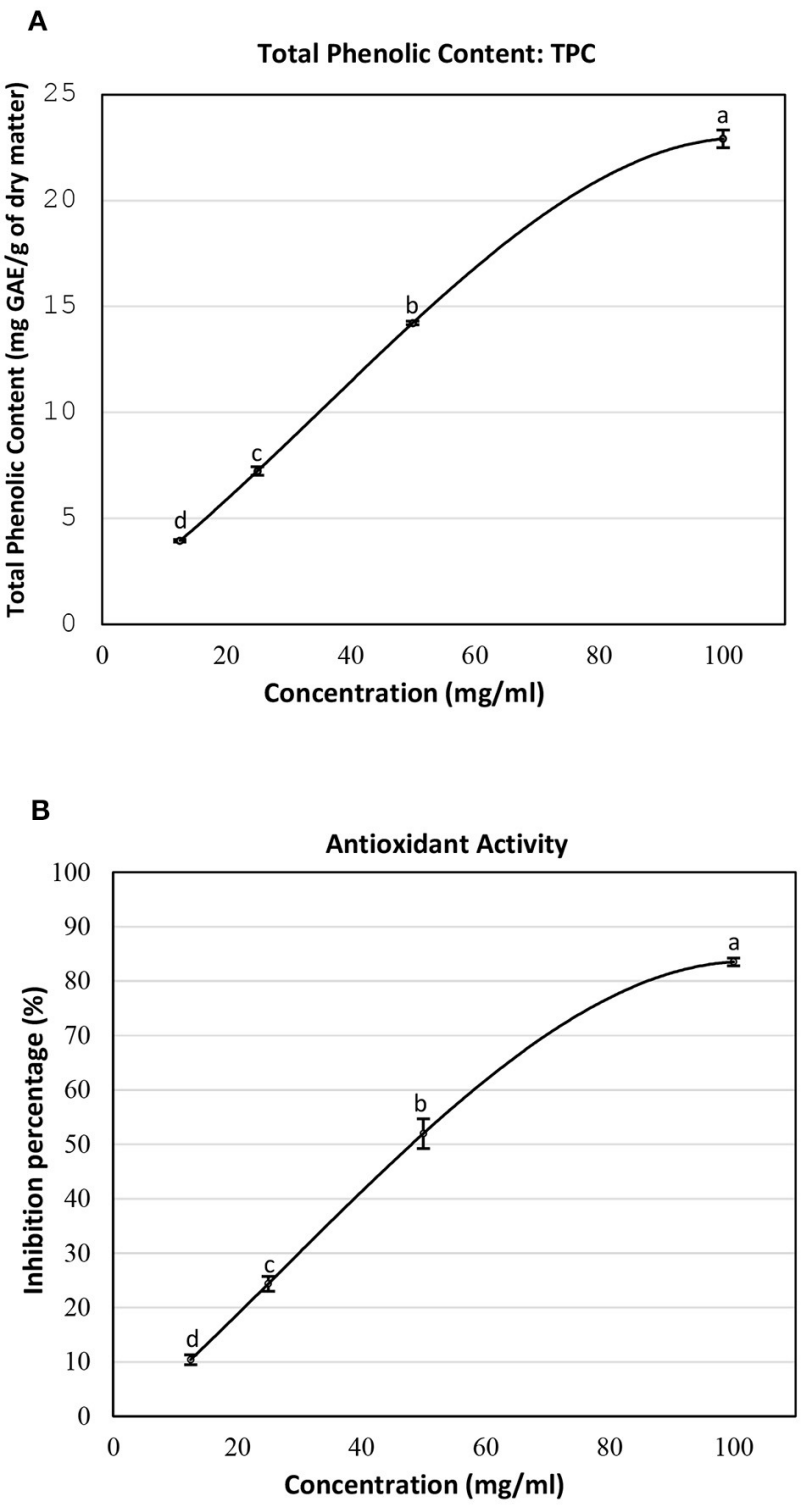
**(A)** Total phenolic content and **(B)** inhibition percentage of the lyophilized SSC extract at different concentrations. Means ± SEM were reported. Means not sharing a similar letter differ significantly (*p* < 0.05).

The total phenolic content and antioxidant activity at different concentrations of the SSC extract are presented in Figure 1. At a concentration of 12.5 mg/ml, the total phenolic content was measured at 3.95 mg GAE/g DM (Figure 1A). This value increased significantly to 7.24 mg GAE/g DM at 25 mg/ml. An additional increase was observed at higher concentrations, with values reaching 14.22 mg GAE/g DM at 50 mg/ml and 22.91 mg GAE/g DM at 100 mg/ml. As is evident, the increasing trend in the total phenolic content suggests a positive correlation between the concentrations of the SSC lyophilized extract and its phenolic richness. In another study, the polyphenol content in the sesame coat was approximately 9.45 mg GAE/g DM when extracted with 70% methanol (13). Besides, a study by El-Roby et al. (11) demonstrated a polyphenol yield of 7.233 mg GAE/g DM from sesame coat when extracted with 80% ethanol. These results are in accordance with the values obtained in this study.

The free radical scavenging activity of the SSC extract was assessed by measuring its antioxidant activity using the DPPH assay. It was assessed in terms of percentage of inhibition (Figure 1B). At a concentration of 12.5 mg/ml of SSC extract, the antioxidant activity was 10.38%. This activity increased significantly to 24.36% at 25 mg/ml. Subsequent elevations were observed at higher concentrations, with antioxidant activities reaching 51.97% at 50 mg/ml and 83.53% at 100 mg/ml. The increasing trend in antioxidant activity suggests a positive correlation between the concentration of the lyophilized extract of SSC and its ability to reduce the DPPH radical. In a comparable study, the inhibition percentage was recorded at 81.29% at a concentration of 180.8 μg/ml for the SSC extract (11). In addition, Elleuch et al. (13) tested the scavenging effect of aqueous 70% ethanol and 70% methanol SSC extracts at a dose of 0.5 mg and it was approximately 94.4% (13). While the antioxidant activity in these studies is closely aligned with our findings, it is important to note that the concentrations of extracts employed in those studies were different. This variation can be attributed to differences in the origin of SSC and the extraction methods used.

Further increasing the concentration of the SSC extract will result in reaching a point of maximum TPC and antioxidant activity, leading to stabilization. These results suggested using the highest concentration of SSC (100 mg/ml) for subsequent methods in this study to achieve optimal outcomes.

### 3.2 Identification of polyphenols by HPLC analysis of the SSC extract

The method was used to detect and quantify phenolic compounds in the SSC extract. The analysis revealed the presence and quantification of five different types of polyphenols (Table 1 and Supplementary Figure 2). The main compounds were catechin, gallic acid, quercetin, *p*-coumaric acid, and caffeic acid. Catechins, members of the flavanol family, have numerous health benefits, such as anticancer, antiobesity, antidiabetic, anticardiovascular, antiinfectious, neuroprotective, and hepatoprotective effects (35). It was the most abundant (2.41 ± 0.45 ppm) and significantly higher than all the other compounds found in the SSC extract (*p* < 0.05). Many studies have proven that the key factors behind the strong effects, such as fighting radicals and bacteria, in sesame seed coat extracts are flavonoids (36, 37). Gallic acid, which belongs to the hydroxybenzoic acid family, has almost the same health benefits as catechins. It is classified as the second most abundant compound in the SSC extract at 1.41 ± 0.07 ppm. However, its concentration was not significantly higher than that of quercetin and p-coumaric acid (*p* < 0.05). Quercetin is a highly abundant polyphenolic bioflavonoid, specifically classified as a flavonol. It is known for its various beneficial biological activities, including antioxidant, anti-inflammatory, anticancer, and antiviral properties (38). It was detected at a concentration of 1.03 ± 0.15 ppm in the SSC, with no significant difference among gallic acid, *p*-Coumaric acid, and caffeic acid (*p* > 0.05). *p*-Coumaric and caffeic acids, classified as hydroxycinnamic acids, have preventive effects against chronic diseases and cancer (39). *p*-Coumaric acid and caffeic acid were detected in the SSC extract (0.99 ± 0.06 ppm and 0.37 ± 0.00 ppm; respectively), with no significant differences between them (*p* > 0.05). El-Roby et al. (11) found similar results, analyzing polyphenols in SSC; however, they were detected at different concentrations (11). This could be due to differences in the sources of the sesame coat. According to the study's findings, the presence of polyphenols in the SSC extract was confirmed by quantifying various phenolic compounds. This discovery suggests that the antibacterial activity of the SSC extract may be positively influenced.

**Table 1:** Polyphenols of the sesame seed coat.

**Compound**	**Concentration (ppm)**	**Chemical structure**
Catechin	2.41 ± 0.45^a^	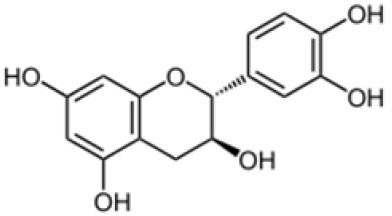
Gallic acid	1.41 ± 0.07^b^	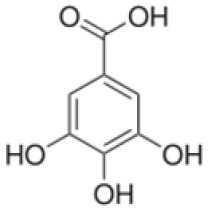
Quercetin	1.03 ± 0.15^bc^	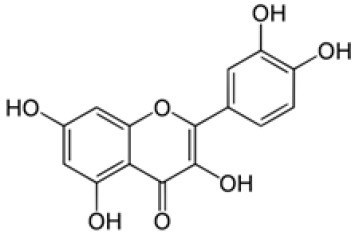
p-Coumaric acid	0.99 ± 0.06^bc^	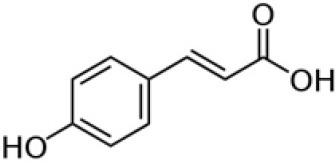
Caffeic acid	0.37 ± 0.00^c^	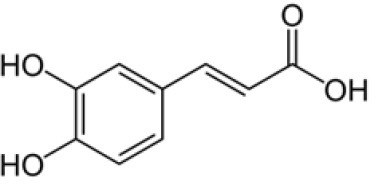

^abc^ Means ± SEM within the column not sharing a similar superscript differ significantly (*p* < 0.05).

### 3.3 Gas chromatography-mass spectrometry analysis of the SSC extract

GC-MS analysis of the SSC extract in the current study showed 7 peaks, including siloxanes, acrylate monomers, fatty acids, and alkanes (Supplementary Figure 1). Table 2 lists the retention times, compound names, and peak areas (%) of the detected compounds. The chemical components identified in the SSC extract include 1.68% dodecamethylcyclohexasiloxane, 1.43% tridecyl acrylate, 5.70% palmitic acid, 64.73% oleic acid, 13.31% stearic acid, 5.52% 2-methyltetracosane, and 7.62% 2-linoleoylglycerol. GC-MS analysis revealed that the SSC extract is predominantly composed of saturated and unsaturated fatty acids, accounting for a combined 83.74%. Oleic acid stands out as the major fatty acid, comprising the highest percentage at 64.73%. This is in accordance with a study conducted by Elleuch et al. (10) on sesame by-products, where the most abundant fatty acid was oleic acid (43%).

**Table 2:** GC-MS analysis of SSC extract.

**Peak number**	**Retention time (min)**	**Area (%)**	**Compound**
1	8.34	1.68	Dodecam ethylcyclohe xasiloxane
2	11.48	1.43	Tridecyl acrylate
3	13.38	5.70	Palmitic acid
4	14.56	64.73	Oleic acid
5	14.67	13.31	Stearic acid
6	14.85	5.52	2-Methyl tetracosane
7	16.20	7.62	2-Linoleoyl glycerol

The primary objective of conducting GC-MS on the SSC extract was to detect the presence of fatty acids, which provided the oily texture necessary for the formulation of the nanoemulsion. By confirming the presence of fatty acids through GC-MS analysis, it was possible to support the rationale behind the formulation of SSC extract within the nanoemulsion. Additionally, the detected fatty acids may play a role in the antibacterial effect. Fatty acids demonstrate detergent properties that elucidate their adverse impact on bacterial cells. This characteristic allows them to engage with the cell membrane, creating temporary or permanent pores of various sizes. At high concentrations, these interactions can cause the lipid bilayer to release components such as membrane proteins or larger segments (40, 41). According to a study conducted by Dilika et al. (42), oleic acid (C18:1), obtained from Helichrysum pedunculatum leaves, has shown antibacterial properties against Gram-positive bacterial strains.

### 3.4 Antibacterial activity of SSC extract

**Figure 2:**
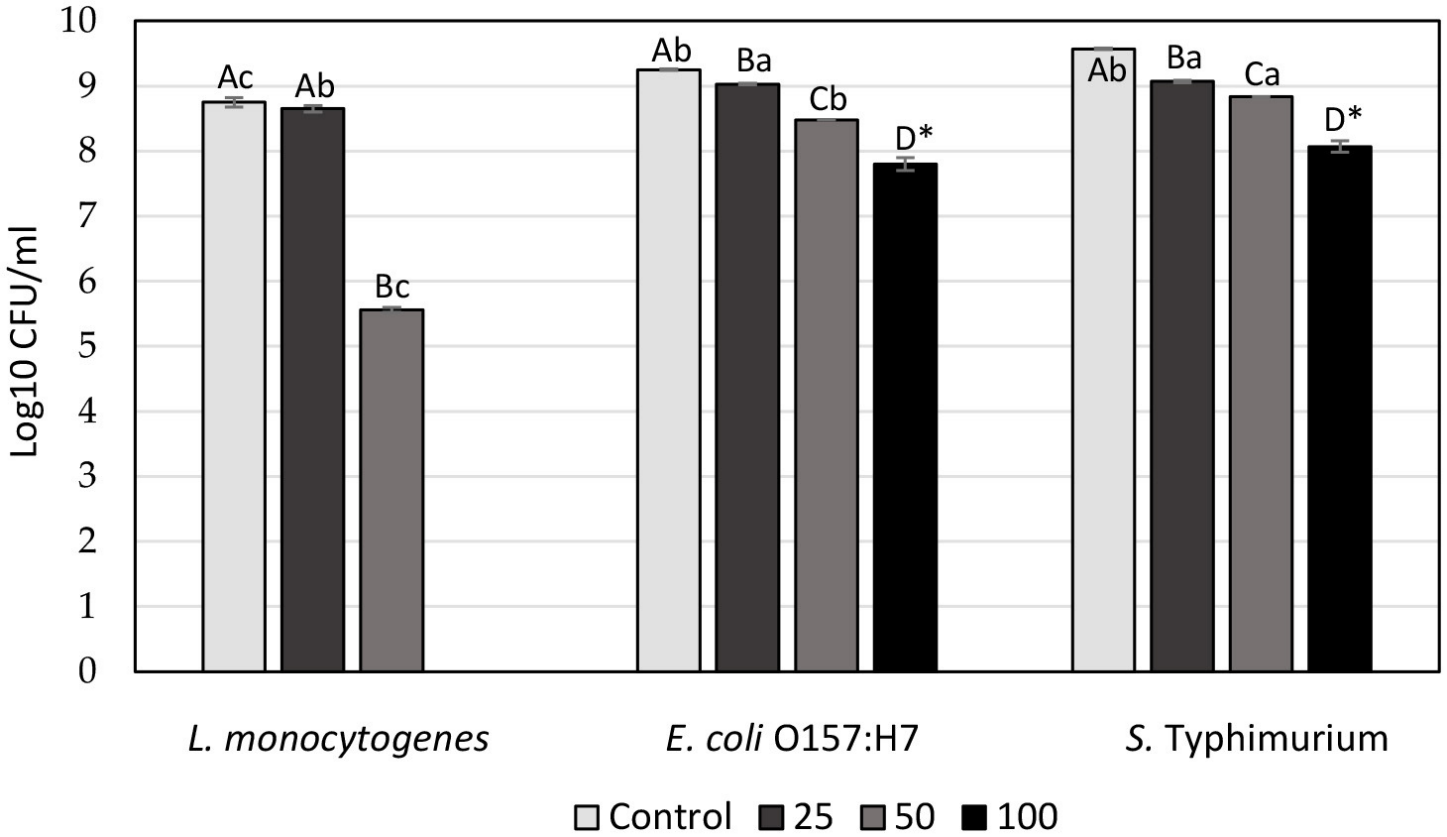
Antibacterial effect of SSC extract at increasing concentration (25, 50, & 100 mg/ml) on the viability of *L. monocytogenes, E. coli* O157:H7, and *S. Typhimurium* (log_10_ CFU/ml). Values are the means of three experiments ± SEM. Values with the same capital letters for the same bacteria and with the same lowercase letters for the same concentration are not significantly different (*p* > 0.05). *Represents independent samples t-test.

Our current study is the first to assess the antibacterial potential of SSC, using the microdilution method. The effect of the SSC extract on the viability of *L. monocytogenes, E. coli*, and *S*. Typhimurium at increasing concentration is presented in Figure 2. A clear trend emerges as the concentration of SSC extract increases, resulting in a corresponding increase in antibacterial capacity. The highest antibacterial effect was observed for *L. monocytogenes* treated with SSC extract at 100 mg/ml, where no viable bacteria were detected. At 100 mg/ml of SSC, there was no significant difference between *E. coli* and *S. Typhimurium* (*p* < 0.05). However, both Gram-negative bacteria had a significant effect with a log reduction of 1.5 (*p* < 0.05). At 50 mg/ml of SSC extract, the antibacterial effectiveness of *L. monocytogenes* (3.2 log reduction) was still significantly higher than *E. coli* and *S*. Typhimurium (*p* < 0.05). However, at a concentration of 25 mg/ml, there was no significant difference between its effect on *L. monocytogenes* (8.65 ± 0.05) and the control (8.75 ± 0.07). Therefore, the MBC of *L. monocytogenes* was below 100 mg/ml, while for *E. coli* and *S*. Typhimurium, the MBC exceeded 100 mg/ml. Most plant extracts show a more potent antibacterial effect against Gram-positive bacteria compared to Gram-negative bacteria (43). This discrepancy may be attributed to the presence of a lipopolysaccharide outer membrane in Gram-negative bacteria, along with porins that regulate the diffusion of substances into the cytosol, contributing to their resistance to antibacterial compounds (44). On the other hand, the Gram-positive bacteria lack an outer membrane.

### 3.5 Characterization of NE-SSC

#### 3.5.1 Particle size distribution and ζ-potential

**Figure 3:**
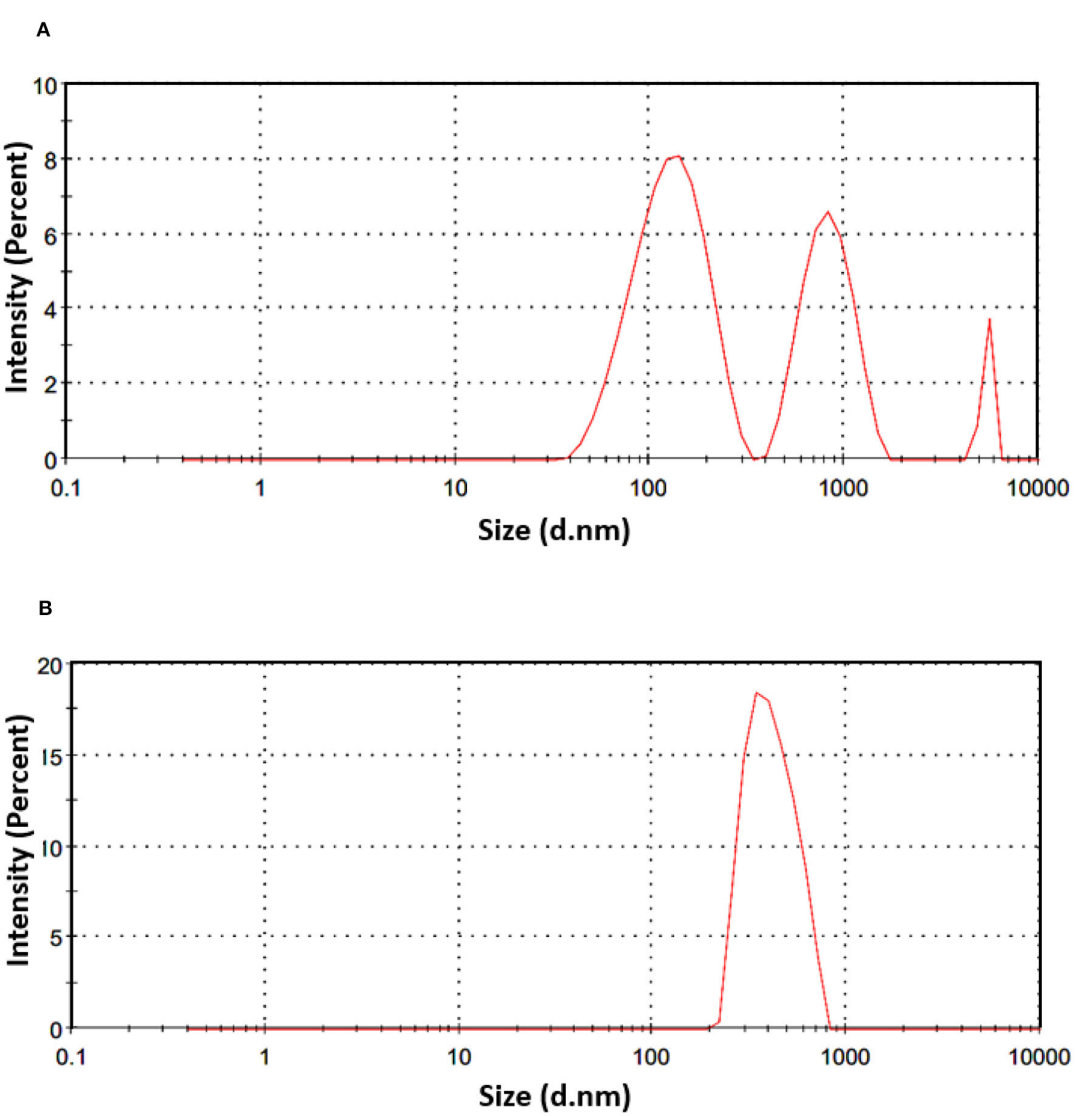
DLS analysis spectra and size distribution by intensity of **(A)** NE-SSC and **(B)** NE.

Dynamic light scattering (DLS) is an imaging method designed for the examination of dried samples showing a sensitivity to dynamic changes in aggregation. The technique indirectly determines particle size by analyzing movement frequency and subsequent modeling. Comparisons with microscopic methods may yield variations in results, given that DLS relies on scattered light analysis. The polydispersity index (PDI), a dimensionless parameter, is used to measure size dispersion, and PDI values below 0.1 indicate a monodisperse state, representing a narrow size distribution. Values above 0.7 are considered too polydisperse for DLS analysis (45). The values of z-average and PDI for NE-SSC and NE are shown in Table 3. The particle size distribution is shown in Figure 3. According to the DLS results, the NE has a peak intensity of 100% and a Z-average value of 1,018 nm, indicating that it is homogeneous. However, the NE-SSC has a bimodal distribution, with the smaller diameter peak exhibiting most of the intensity of 60.7% and a Z-average value of 273.5 nm. Furthermore, NE-SSC has a PDI of 0.585, which confirms that it is monodisperse. This bimodal distribution confirms the presence of SSC in the formed NE-SSC. After the successful preparation of NE-SSC was confirmed, it was necessary to study the stability of high-throughput production of this nanoemulsion. The surface charge of the formed NE-SSC was evaluated using the Zeta potential (ζ-potential). Table 3 shows the ζ-potential values of NE-SSC and NE. The data in Table 3 reveal that the value of ζ-potential for NE is 1.04 mV, which increases to 8.18 mV for NE-SSC. These values indicate that NE-SSC exhibits greater stability compared to NE. The higher value of ζ-potential plays an important role in increasing the electrostatic repulsive forces between the surfaces of the particles, which leads to a good distribution of the nanoemulsion NE-SSC (46).

**Table 3:** Size parameters obtained by the DLS and zeta potential for NE-SSC and NE.

**Particle**	**Z-average size distribution diameter (nm)**	**PDI**	**Zeta potential (mV)**	**Peak intensity (%)**
NE-SSC	273.5	0.585	8.18	60.7/34.7/4.6
NE	1,018	1	1.04	100

#### 3.5.2 SEM

**Figure 4:**
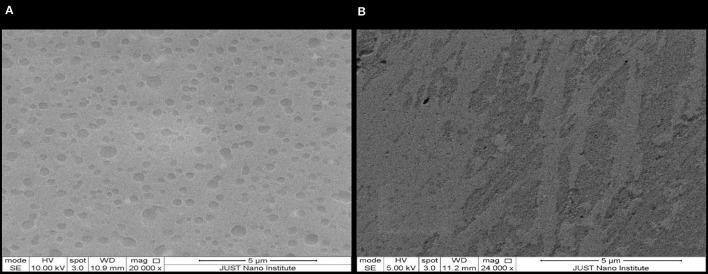
SEM images for **(A)** NE-SSC and **(B)** NE.

Generally, image acquisition through scanning electron microscopy proved to be challenging. Multiple attempts were made with various imaging and sample preparation conditions for each sample before identifying the optimal conditions. This replication process was necessary to ensure the production of high-quality images, highlighting the complexity and precision required in the use of scanning electron microscopy for this particular study. Figure 4A shows randomly oriented nano-sized spheres of NE-SSC with various shapes and sizes. However, Figure 4B illustrates quite a different morphology for NE compared to NE-SSC. We observe a rough and uneven surface in Figure 4B. This demonstrates the efficacy of the nanoemulsion formulation in comparison to the control.

### 3.6 Alterations in the morphology of bacterial cells

**Figure 5:**
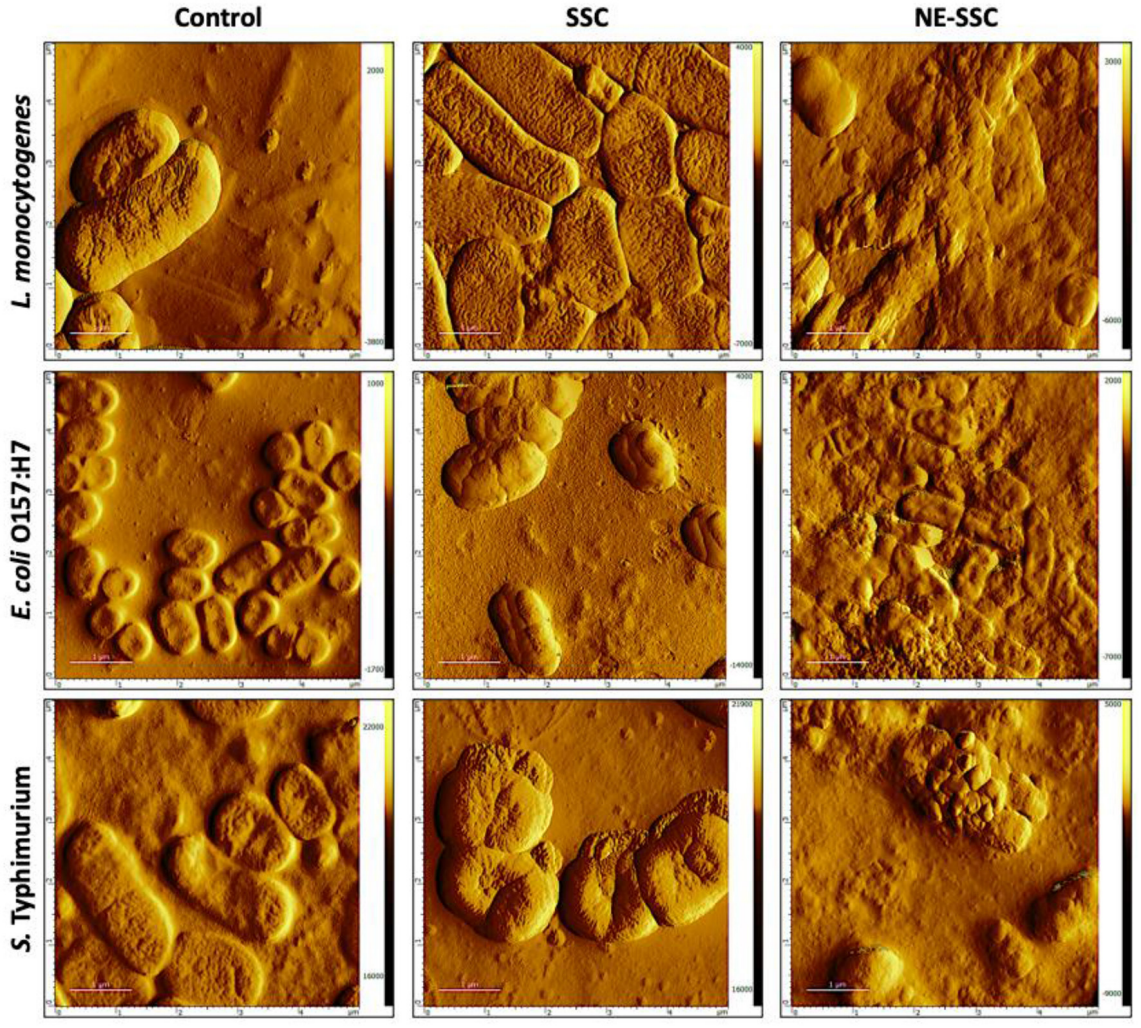
Atomic force microscopy (AFM) images of *L. monocytogenes, E. coli* O157:H7, and *S. Typhimurium* control and treated cells. Scan area 5 μm × 5 μm.

Bacterial imaging was carried out using atomic force microscopy (AFM), a potent tool for studying microbial surfaces and structural alterations on the nanometer scale (47). In this study, the non-contact mode was employed, where the cantilever maintained a distance of tens to hundreds of angstroms (Å) away from the sample, only vibrating in proximity to it (48). This approach protects both the sample and the tip from potential damage, making it advantageous over contact mode (49). The main purpose of incorporating AFM in this study was to analyze the morphological changes that occur in bacterial cells. The AFM images of control and treated bacterial cells are shown in Figure 5. The control images revealed smooth bacterial surfaces with typical rod-shaped morphology for *L. monocytogenes* and Gram-negative bacteria (i.e., *E. coli* O157:H7 and *S. Typhimurium*). However, when treated with SSC (100 mg/ml), the bacteria showed surface protrusions, stiffness, and swelling, while *S. Typhimurium* only showed some roughness on the surface. Upon treatment with NE-SSC, the bacteria had distorted shapes and indentations. This observation may imply a synergistic effect for the antibacterial activity of SSC when it is encapsulated within a nanoemulsion.

### 3.7 Antibacterial activity of SSC extract in milk

Considering that food matrices consist of diverse food ingredients, the addition of an antibacterial can lead to various molecular interactions. These interactions may influence antibacterial behavior within the food matrix differently than in microbiological media (50, 51). In this study, sterilized milk stored at 4 and 10°C for 7 days was employed as a food model system (*in situ*) to assess the effectiveness of SSC at a concentration of 100 mg/ml. Table 4 illustrates the impact of SSC against *L. monocytogenes* and *E. coli* O157:H7 on days 0, 1, 3, and 7 in milk subjected to incubation at both 4 and 10°C, comparing control samples with those enriched with SSC extract.

**Table 4:** Effect of SSC on *L. monocytogenes* and *E. coli* O157:H7 in milk stored at 4 and 10°C.

**Temperature**	**Day**	***L. monocytogenes***			***E. coli*** **O157:H7**			**Between bacteria**	
		**Control**	**Extract**		**Control**	**Extract**		**Control**	**Extract**
				***p-value***			***p-value***	***p-value***	***p-value***
4°C	0	4.52 ± 0.02^d^	4.52 ± 0.02^d^	1	4.06 ± 0.06^c^	4.06 ± 0.06^b^	1	0.002^**^	0.002^**^
	1	5.53± 0.08^c^	4.29 ± 0.15^ab^	0.002^**^	3.41 ± 0.06^d^	3.46 ± 0.24^c^	0.852	< 0.001^***^	0.041^*^
	3	7.34 ± 0.02^b^	4.00 ± 0.00^bc^	< 0.001^***^	5.69 ± 0.03^b^	4.58 ± 0.16^a^	0.002^**^	< 0.001^***^	0.021^*^
	7	7.96 ± 0.08^a^	3.74 ± 0.14^c^	< 0.001^***^	7.87 ± 0.00^a^	4.61 ± 0.14^a^	0.002^**^	0.302	0.011^*^
p-value		< 0.001^***^	0.003^**^		< 0.001	0.003			
10°C	0	4.68 ± 0.10^d^	4.68 ± 0.10^a^	1	3.79 ± 0.16^c^	3.79 ± 0.12^c^	1	0.005^**^	0.005^**^
	1	5.85 ± 0.10^c^	4.81 ± 0.02^a^	0.007^**^	4.32 ± 0.12^c^	3.58 ± 0.16^c^	0.02^*^	< 0.001^***^	0.002^**^
	3	7.11 ± 0.05^b^	3.93 ± 0.07^b^	< 0.001^***^	6.89 ± 0.39^b^	4.73 ± 0.09^b^	0.026^**^	0.643	0.002^**^
	7	8.24 ± 0.07^a^	3.79 ± 0.26^b^	< 0.001^***^	8.37 ± 0.06^a^	7.47 ± 0.09^a^	0.001^**^	0.219	< 0.001^***^
p-value		< 0.001^***^	0.002^**^		< 0.001^***^	< 0.001^***^			

Values are the means (log10 CFU/ml) of two experiments ± SEM.

Means within the column not sharing a similar superscript differ significantly (^*^p < 0.05, ^**^p < 0.01, and ^***^p < 0.001, where ^*^, ^**^, and ^***^ denote levels of significance difference).

The results revealed a significant inhibitory effect of SSC extract when added to milk against *L. monocytogenes* at 4 and 10°C (*p* < 0.05). At incubation temperatures of 4 and 10°C, reductions of 4.2 and 4.5 log CFU/ml, respectively, were observed for *L. monocytogenes* at the end of the storage period (Table 4 and Supplementary Table 1). Additionally, SSC exhibited a significant inhibitory effect against *E. coli* O157:H7 at 4°C, resulting in a log CFU/ml 3.3 after 7 days (*p* < 0.05). However, at 10°C, a relatively lower reduction of approximately 0.9 log CFU/ml was observed for *E. coli* O157:H7 (Table 4).

Clearly, the inhibitory effects of the SSC extract were more pronounced against *L. monocytogenes* when subjected to the optimal growth temperature of 37°C, except for *E. coli* O157:H7 at 4°C. This phenomenon can be attributed to the increased metabolic activity exhibited by bacterial cells at their optimal growth temperatures. Specifically, at 37°C, the presence of SSC extract at a concentration of 100 mg/ml effectively inhibited the growth of *L. monocytogenes* (Figure 2). On the contrary, at lower temperatures of 4 and 10°C, the impact of the SSC extract was limited to a reduction in bacterial cell count. This increased efficacy can be associated with the increased antibacterial activity observed at elevated temperatures (52, 53), which is likely related to the increased active state of bacterial cells, resulting in elevated growth and death rates at elevated temperatures (54). In contrast, lower temperatures tend to slow the growth rate of bacterial cells, making them less susceptible to the action of the antimicrobial agent (55). Furthermore, the inhibition effect of SSC against *L. monocytogenes* in milk was lower compared to that in broth. This discrepancy is attributed to the ability of milk to supply ample nutrients, which promotes bacterial growth (56). Typically, in antimicrobial challenge assessments, achieving a 2-log reduction in the target microbial strain holds significant practical implications. However, variables such as the initial inoculum level can affect the ultimate reduction outcome (57).

### 3.8 Antibacterial activity of NE-SSC in milk

Table 5 shows a comparison of the effect of different concentrations of NE and NE-SSC on *L. monocytogenes* and *E. coli* O157:H7 in milk stored at 4 and 10°C. The antibacterial impact of 3% NE on *L. monocytogenes* and *E. coli* O157:H7 was found to be statistically insignificant compared to 0% NE on day 7 at both 4 and 10°C (*p* > 0.05), except for a minimal reduction of 0.33 log CFU/ml observed for *E. coli* O157:H7 at 10°C (Table 5). However, using 7% NE, a significant effect against both bacteria was observed at 4 and 10°C on day 7, ranging between 0.12 and 2.19 log CFU/ml reductions compared to 0% NE (*p* < 0.05). The antibacterial efficacy of NE also increased at 10% concentration at both incubation temperatures on day 7, with reductions ranging between 0.58 and 3.76 log CFU/ml (*p* < 0.05).

**Table 5:** Comparison of the antibacterial effect of different concentrations of NE and NE-SSC on *L. monocytogenes* and *E. coli* O157:H7 in milk stored at 4 and 10°C.

**Bacteria**	**Temperature**	**Day**	**NE**					**NE-SCC**				
			**0%**	**3%**	**7%**	**10%**		**0%**	**3%**	**7%**	**10%**	
							***p-value***					***p-value***
*L. monocytogenes*	4°C	0	4.41 ± 0.06	4.41 ± 0.06	4.41 ± 0.06	4.41 ± 0.06	1	4.41 ± 0.06	4.41 ± 0.06	4.41 ± 0.06	4.41 ± 0.06	1
		1	5.50 ± 0.06^a^	5.48 ± 0.04^a^	5.16 ± 0.08^b^	4.92 ± 0.04^c^	< 0.001^***^	5.50 ± 0.06^a^	5.39 ± 0.02^a^	5.03 ± 0.16^b^	4.63 ± 0.02^c^	< 0.001^***^
		3	7.53 ± 0.05^a^	7.38 ± 0.07^a^	6.51 ± 0.03^b^	5.45 ± 0.08^c^	< 0.001^***^	7.53 ± 0.05^a^	7.02 ± 0.06^b^	5.59 ± 0.02^c^	5.00 ± 0.03^d^	< 0.001^***^
		7	8.09 ± 0.04^a^	8.06 ± 0.04^a^	7.52 ± 0.18^b^	6.88 ± 0.10^c^	< 0.001^***^	8.09 ± 0.04^a^	8.13 ± 0.06^a^	7.14 ± 0.10^b^	5.46 ± 0.09^c^	< 0.001^***^
	10°C	0	4.41 ± 0.06	4.41 ± 0.06	4.41 ± 0.06	4.41 ± 0.06	1	4.41 ± 0.06	4.41 ± 0.06	4.41 ± 0.06	4.41 ± 0.06	1
		1	6.07 ± 0.11^a^	5.58 ± 0.07^b^	5.30 ± 0.02^c^	5.04 ± 0.03^d^	< 0.001^***^	6.07 ± 0.11^a^	5.26 ± 0.05^b^	4.68 ± 0.01^c^	4.58 ± 0.05^c^	< 0.001^***^
		3	7.79 ± 0.12^a^	7.33 ± 0.12^b^	6.43 ± 0.08^c^	5.90 ± 0.20^d^	< 0.001^***^	7.79 ± 0.12^a^	7.12 ± 0.08^b^	5.56 ± 0.14^c^	5.11 ± 0.05^d^	< 0.001^***^
		7	8.04 ± 0.02^b^	8.24 ± 0.05^a^	7.92 ± 0.04^b^	7.46 ± 0.03^c^	< 0.001^***^	8.04 ± 0.02^a^	8.21 ± 0.11^a^	7.61 ± 0.07^b^	6.23 ± 0.05^c^	< 0.001^***^
*E. coli* O157:H7	4°C	0	3.99 ± 0.02	3.99 ± 0.02	3.99 ± 0.02	3.99 ± 0.02	1	3.99 ± 0.02	3.99 ± 0.02	3.99 ± 0.02	3.99 ± 0.02	1
		1	4.37 ± 0.05	4.44 ± 0.12	4.15 ± 0.16	4.10 ± 0.06	0.124	4.37 ± 0.05	4.34 ± 0.11	4.15 ± 0.17	3.89 ± 0.22	0.173
		3	6.02 ± 0.04^a^	5.69 ± 0.11^b^	4.87 ± 0.08^c^	4.48 ± 0.10^d^	< 0.001^***^	6.02 ± 0.04^a^	5.60 ± 0.00^b^	4.76 ± 0.03^c^	4.14 ± 0.04^d^	< 0.001^***^
		7	7.66 ± 0.03^a^	8.09 ± 0.21^a^	5.47 ± 0.54^b^	4.25 ± 0.01^c^	< 0.001^***^	7.66 ± 0.03^a^	7.58 ± 0.04^a^	5.24 ± 0.26b	3.72 ± 0.07^c^	< 0.001^***^
	10°C	0	3.99 ± 0.02	3.99 ± 0.02	3.99 ± 0.02	3.99 ± 0.02	1	3.99 ± 0.02	3.99 ± 0.02	3.99 ± 0.02	3.99 ± 0.02	1
		1	5.11 ± 0.06^a^	4.73 ± 0.04^b^	4.33 ± 0.03^c^	4.12 ± 0.07^d^	< 0.001^***^	5.11 ± 0.06^a^	3.92 ± 0.26^b^	3.57 ± 0.16^b^	3.99 ± 0.05^b^	< 0.001^***^
		3	7.05 ± 0.06^a^	6.95 ± 0.03^a^	5.85 ± 0.00^b^	4.45 ± 0.03^c^	< 0.001^***^	7.05 ± 0.06^a^	6.67 ± 0.05^b^	4.87 ± 0.03^c^	4.27 ± 0.05^d^	< 0.001^***^
		7	8.87 ± 0.02^a^	8.54 ± 0.11^b^	8.01 ± 0.04^c^	5.11 ± 0.06^d^	< 0.001^***^	8.87 ± 0.02^a^	8.23 ± 0.01^b^	5.20 ± 0.10^c^	4.14 ± 0.07^d^	< 0.001^***^

Values are means (log10 CFU/ml) of three experiments ± SEM.

Means within row not sharing a similar superscript differ significantly (^*^p < 0.05, ^**^p < 0.01, and ^***^p < 0.001, where ^*^, ^**^, and ^***^ denote levels of significance difference).

Row-wise comparisons were performed using ANOVA with DMRT *post-hoc*.

The antibacterial influence of 3% NE-SSC on both *L. monocytogenes* and *E. coli* O157:H7 was statistically insignificant compared to 0% NE-SSC at the end of the storage period at 4 and 10°C (*p* > 0.05). However, a notable reduction of 0.64 log CFU/ml was observed for *E. coli* O157:H7 at 10°C (Table 5). In contrast, at 7% NE-SSC, a significant antibacterial effect against both bacteria were evident at 4 and 10°C on day 7, resulting in reductions ranging from 0.43 to 3.67 log CFU/ml compared to 0% NE-SSC (*p* < 0.05). The antibacterial efficacy of NE-SSC was further increased at a 10% concentration at both incubation temperatures on day 7, yielding reductions ranging between 1.81 and 4.73 log CFU/ml (*p* < 0.05).

In summary, concentrations of 7% and 10% for both NE and NE-SSC demonstrated significant efficacy at 4 and 10°C in reducing viable counts of *L. monocytogenes* and *E. coli* O157:H7 after incubation for 7 days.

Table 6 outlines the differences in the antibacterial effect between NE and NE-SSC against *L. monocytogenes* and *E. coli* O157:H7 in milk over 7 days of storage at 4 and 10°C. At 3% of both NE and NE-SSC, there were no significant differences in logarithmic count between them after 7 days at 4 and 10°C (*p* > 0.05) (Table 6 and Supplementary Table 2). However, at a concentration of 7%, there was a significant difference in effectiveness between NE (7.92, 8.01 log CFU/ml) and NE-SSC (7.61, 5.20 log CFU/ml) against *L. monocytogenes* and *E. coli* O157:H7 at 10°C at the end of the incubation period, respectively (*p* < 0.05). This resulted in a difference of 0.31 and 2.81 log CFU/ml between the effects of 7% NE and NE-SSC on *L. monocytogenes* and *E. coli* O157:H7 at 10°C, respectively.

**Table 6:** Difference between the antibacterial effect of NE and NE-SSC on *L. monocytogenes* and *E. coli* O157:H7 in milk stored at 4 and 10°C.

**Bacteria**	**Temperature**	**Day**	**0%**			**3%**			**7%**			**10%**		
			**NE**	**NE-SCC**		**NE**	**NE-SCC**		**NE**	**NE-SCC**		**NE**	**NE-SCC**	
					***p-value***			***p-value***			***p-value***			***p-value***
*L. monocytogenes*	4°C	0	4.41 ± 0.06^d^	4.41 ± 0.06^d^	1	4.41 ± 0.06^d^	4.41 ± 0.06^d^	1	4.41 ± 0.06^d^	4.41 ± 0.06^d^	1	4.41 ± 0.06^d^	4.41 ± 0.06^d^	1
		1	5.50 ± 0.06^c^	5.50 ± 0.06^c^	1	5.48 ± 0.04^c^	5.39 ± 0.02^c^	0.134	5.16 ± 0.08^c^	5.03 ± 0.16^c^	0.503	4.92 ± 0.04^c^	4.63 ± 0.02^c^	0.003^**^
		3	7.53 ± 0.05^b^	7.53 ± 0.05^b^	1	7.38 ± 0.07^b^	7.02 ± 0.06^b^	0.017^*^	6.51 ± 0.03^b^	5.59 ± 0.03^b^	< 0.001^***^	5.45 ± 0.08^b^	5.00 ± 0.04^b^	0.005^**^
		7	8.09 ± 0.04^a^	8.09 ± 0.04^a^	1	8.06 ± 0.03^a^	8.13 ± 0.07^a^	0.437	7.52 ± 0.18^a^	7.14 ± 0.10^a^	0.135	6.88 ± 0.10^a^	5.46 ± 0.09^a^	< 0.001^***^
	*p-value*		< 0.001^***^	< 0.001^***^		< 0.001^***^	< 0.001^***^		< 0.001^***^	< 0.001^***^		< 0.001^***^	< 0.001^***^	
	10°C	0	4.41 ± 0.06^c^	4.41 ± 0.06^c^	1	4.41 ± 0.06^d^	4.41 ± 0.06^d^	1	4.41 ± 0.06^d^	4.41 ± 0.06^d^	1	4.41 ± 0.06^d^	4.41 ± 0.06^d^	1
		1	6.07 ± 0.11^b^	6.07 ± 0.11^b^	1	5.58 ± 0.07^c^	5.26 ± 0.05^c^	0.021^*^	5.30 ± 0.02^c^	4.68 ± 0.01^c^	< 0.001^***^	5.04 ± 0.03^c^	4.58 ± 0.06^c^	0.001^*^
		3	7.79 ± 0.12^a^	7.79 ± 0.12^a^	1	7.33 ± 0.12^b^	7.12 ± 0.08^b^	0.213	6.43 ± 0.08^b^	5.56 ± 0.14^b^	0.005^**^	5.90 ± 0.20^b^	5.11 ± 0.05^b^	0.019^*^
		7	8.04 ± 0.02^a^	8.04 ± 0.02^a^	1	8.24 ± 0.05^a^	8.21 ± 0.11^a^	0.828	7.92 ± 0.05^a^	7.61 ± 0.07^a^	0.018^*^	7.46 ± 0.03^a^	6.23 ± 0.05^a^	< 0.001^***^
	*p-value*		< 0.001^***^	< 0.001^***^		< 0.001^***^	< 0.001^***^		< 0.001^***^	< 0.001^***^		< 0.001^***^	< 0.001^***^	
*E. coli* O157:H7	4°C	0	3.99 ± 0.02^d^	3.99 ± 0.02^d^	1	3.99 ± 0.02^d^	3.99 ± 0.02^d^	1	3.99 ± 0.02^b^	3.99 ± 0.02^b^	1	3.99 ± 0.02^c^	3.99 ± 0.02	1
		1	4.37 ± 0.05^c^	4.37 ± 0.05^c^	1	4.44 ± 0.12^c^	4.34 ± 0.11^c^	0.534	4.15 ± 0.16^b^	4.15 ± 0.17^b^	0.979	4.10 ± 0.06^bc^	3.89 ± 0.22	0.399
		3	6.02 ± 0.05^b^	6.02 ± 0.05^b^	1	5.69 ± 0.11^b^	5.60 ± 0.00^b^	0.461	4.87 ± 0.08^ab^	4.76 ± 0.03^a^	0.258	4.48 ± 0.10^a^	4.14 ± 0.04	0.034^*^
		7	7.66 ± 0.03^a^	7.66 ± 0.03^a^	1	8.09 ± 0.21^a^	7.58 ± 0.04^a^	0.134	5.47 ± 0.54^a^	5.24 ± 0.26^a^	0.722	4.25 ± 0.01^b^	3.72 ± 0.07	0.002^**^
	*p-value*		< 0.001^***^	< 0.001^***^		< 0.001^***^	< 0.001^***^		0.021^*^	0.002^**^		0.002^**^	0.152	
	10°C	0	3.99 ± 0.02^d^	3.99 ± 0.02^d^	1	3.99 ± 0.02^d^	3.99 ± 0.02^c^	1	3.99 ± 0.02^d^	3.99 ± 0.02^d^	1	3.99 ± 0.02^c^	3.99 ± 0.02^b^	1
		1	5.11 ± 0.06^c^	5.11 ± 0.06^c^	1	4.73 ± 0.04^c^	3.92 ± 0.26^c^	0.035^*^	4.33 ± 0.03^c^	3.57 ± 0.16^c^	0.009^**^	4.12 ± 0.07^c^	3.99 ± 0.05^b^	0.212
		3	7.05 ± 0.06^b^	7.05 ± 0.06^b^	1	6.95 ± 0.03^b^	6.67 ± 0.05^b^	0.010^*^	5.85 ± 0.00^b^	4.87 ± 0.03^b^	< 0.001^***^	4.45 ± 0.03^b^	4.27 ± 0.05^a^	0.042^*^
		7	8.87 ± 0.02^a^	8.87 ± 0.02^a^	1	8.54 ± 0.11^a^	8.23 ± 0.01^a^	0.055	8.01 ± 0.04^a^	5.20 ± 0.10^a^	< 0.001^***^	5.11 ± 0.06^a^	4.14 ± 0.07^ab^	< 0.001^***^
	*p-value*		< 0.001^***^	< 0.001^***^		< 0.001^***^	< 0.001^***^		< 0.001^***^	< 0.001^***^		< 0.001^***^	0.010^*^	

Values are means (log10 CFU/ml) of three experiments ± SEM.

Means within the column not sharing a similar superscript differ significantly (*p* < 0.05).

Column-wise comparisons were performed using ANOVA with DMRT *post-hoc*.

Row-wise comparisons were made using independent samples *t*-test.

At a concentration of 10%, there was a significant difference between NE and NE-SSC against *L. monocytogenes* and *E. coli* O157:H7 at 4 and 10°C after 7 days (*p* < 0.05). The variation in counts ranged between 0.53 and 1.42 log CFU/ml for the effects of 10% NE and NE-SSC against both bacteria at the end of the study period at both incubation temperatures. In all instances of significant differences at all concentrations, the addition of NE-SSC to milk exhibited lower bacterial counts than that of NE. At a 10% concentration of NE-SSC, the reduction in the number of *L. monocytogenes* was 2.6 and 1.81 log CFU/ml at 4 and 10°C, respectively. Regarding *E. coli* O157:H7, the reductions were 3.94 and 4.73 log CFU/ml at 10% NE-SSC at 4 and 10°C, respectively. The SSC extract exhibited a capacity to reduce bacterial counts of *L. monocytogenes* and *E. coli* O157:H7, achieving reductions ranging from 0.9 to 4.5 log CFU/ml under identical conditions on day 7. In contrast, NE-SSC at a concentration of 10% demonstrated effectiveness in reducing bacterial counts for both strains at the end of the incubation period, achieving reductions in the range of 1.81 to 4.73 log CFU/ml. This suggests that the inclusion of SSC into the NE enhanced its antibacterial efficacy, demonstrating a synergistic effect and affirming the effectiveness of nanoemulsion formation.

## 4 Conclusion

The SSC stands out as a valuable agricultural by-product with considerable potential for valorization. This study revealed that SSC has a total phenolic content of 22.91 mg GAE/g of DM and an antioxidant activity of 83.53% at 100 mg/ml. HPLC analysis identified five distinct polyphenols in SSC, with catechin registering the highest concentration at 2.41 ppm. Furthermore, GC-MS analysis revealed that the SSC extract is predominantly made up of a mixture of saturated and unsaturated fatty acids, comprising a cumulative 83.74%. Remarkably, SSC demonstrated significant antibacterial effects against *L. monocytogenes* and *E. coli* O157:H7 in both broth and milk. The formation of a nanoemulsion played a crucial role in enhancing the antibacterial activity of SSC, with 10% NE-SSC incorporation in milk exhibiting a pronounced effect against *L. monocytogenes* and *E. coli* O157:H7. Our findings highlight the potential of the sesame seed coat as a natural preservative and antibacterial agent. This innovative approach opens the door to diverse applications in the future, including the potential use of SSC nanoemulsions in active food packaging films aimed at preserving numerous perishable food products. While our study found that the formation of a nanoemulsion enhanced the antibacterial activity of SSC, further research is needed to optimize the formulation and explore its potential applications. Additionally, the economic and technical viability of utilizing SSC as a natural preservative and antibacterial agent in the food industry has promising potential. However, more research is needed to explore the feasibility of large-scale production and commercialization of SSC extracts and nanoemulsions. Overall, our findings highlight the potential of the sesame seed coat as a natural preservative and antibacterial agent, suggesting that it has the potential to be a natural and effective alternative to synthetic preservatives in the food industry. This innovative approach opens the door to diverse applications in the future, including the potential use of SSC nanoemulsions in active food packaging films aimed at preserving numerous perishable food products, and further research in this area is warranted.

## Data availability statement

The original contributions presented in the study are included in the article/Supplementary material, further inquiries can be directed to the corresponding author.

## Author contributions

SK: Conceptualization, Methodology, Formal analysis, Investigation, Resources, Writing—original draft, Writing—review & editing, Visualization. MK: Conceptualization, Methodology, Validation, Writing—review & editing, Visualization, Supervision, Project administration. TO: Conceptualization, Methodology, Validation, Investigation, Resources, Writing—review & editing, Project administration. BA: Methodology, Resources, Writing—review & editing. AA-N: Methodology, Resources, Writing—review & editing. NL: Writing—review & editing. NE: Conceptualization, Methodology, Validation, Formal analysis, Funding acquisition, Investigation, Resources, Writing—review & editing, Visualization, Supervision, Project administration.
