# Supplementary material for: Valorization of sesame seed coat waste: phenolic composition, antibacterial efficacy, and nanoemulsion encapsulation for food preservation
Source: Front Nutr. 2024 Jun 14;11:1405708. doi: 10.3389/fnut.2024.1405708 (PMC11211370; doi:10.3389/fnut.2024.1405708)
Supplement: Supplementary file 1 [file Data_Sheet_1.docx]

Supplementary Material

**(A)**


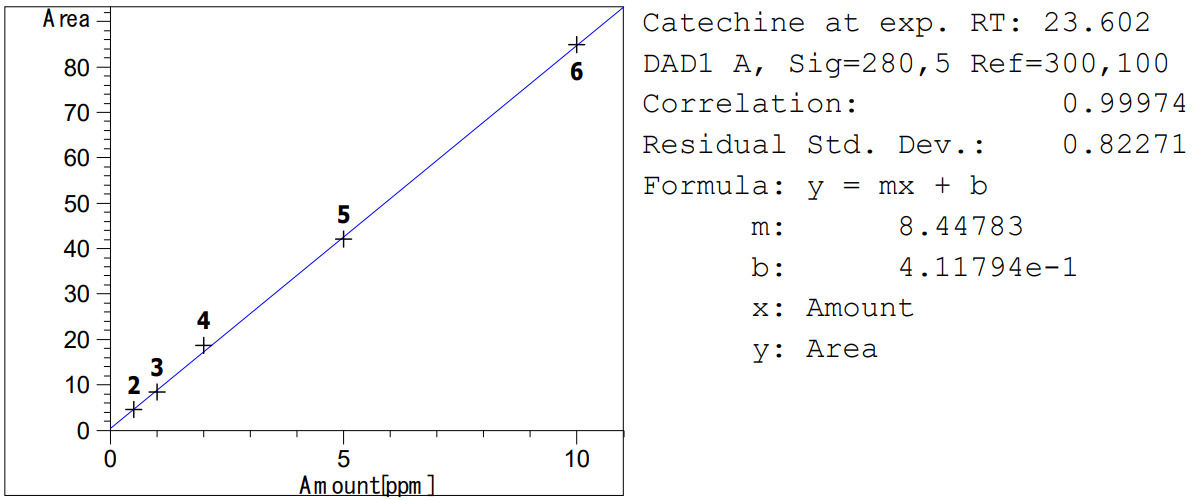


**(B)**


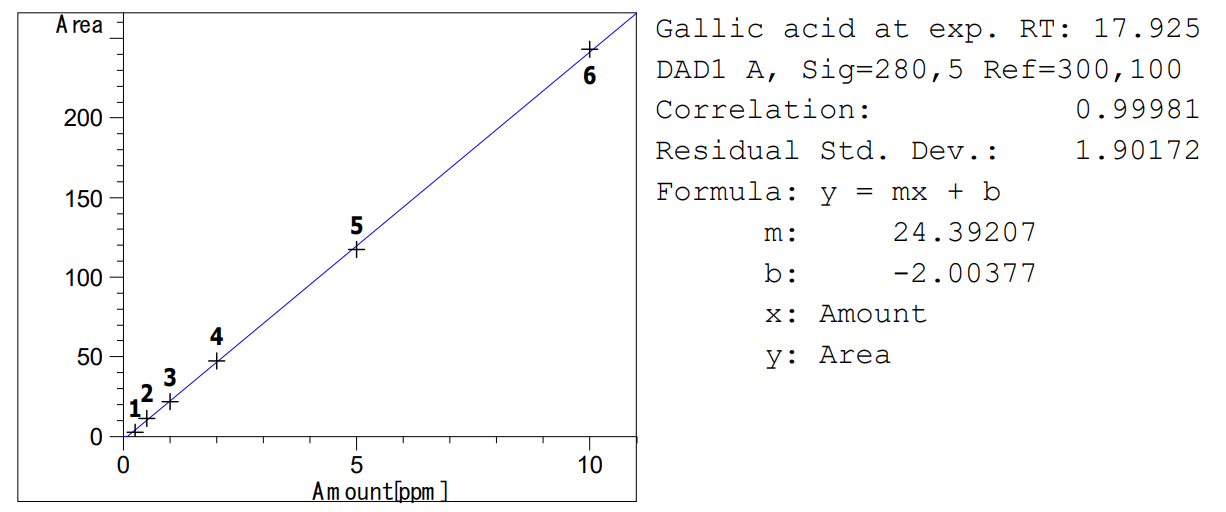


**(C)**


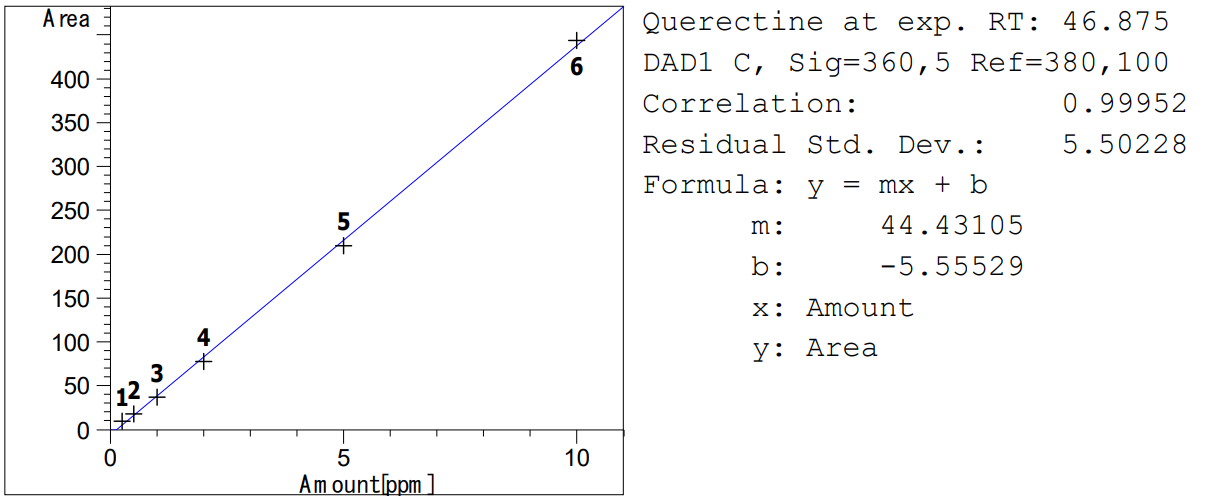


**(D)**


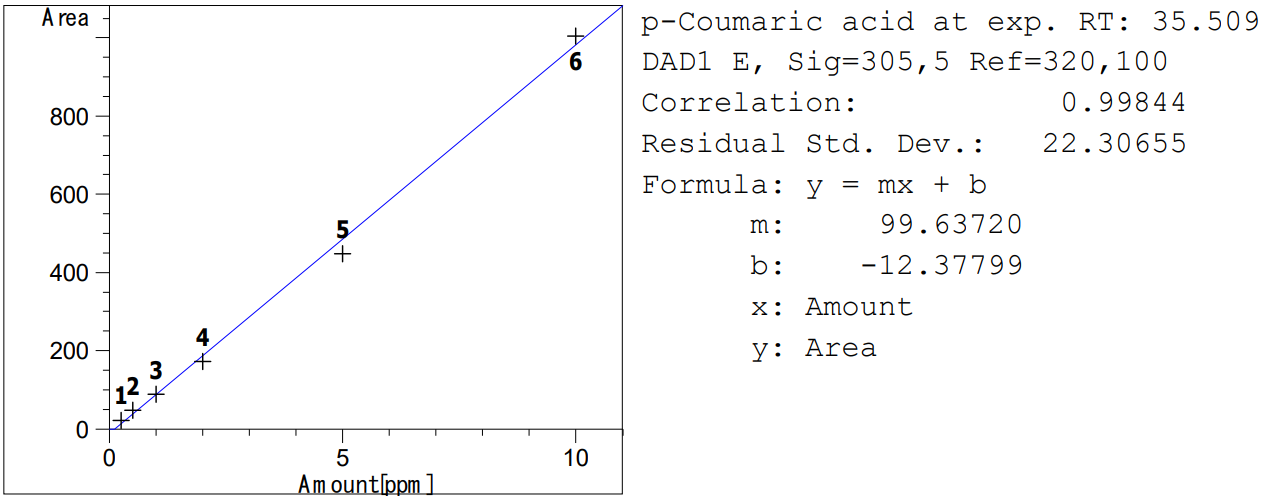


**(E)**


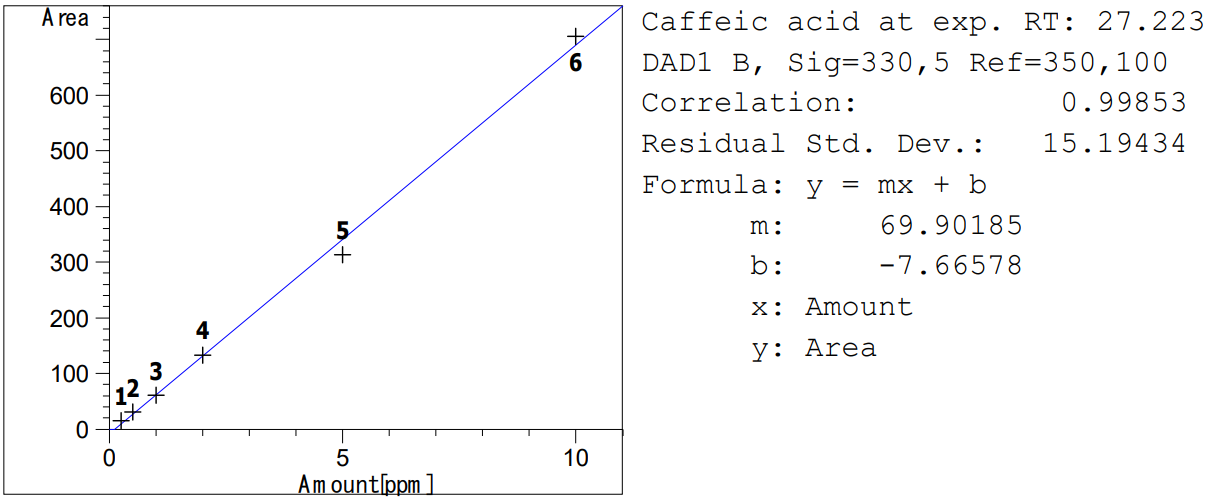


Figure S1: Polyphenols’ standard calibration curves: **(A)** catechin, **(B)** gallic acid, **(C)** quercetin, **(D)** p-Coumaric acid, and **(E)** caffeic acid.

**(A)**


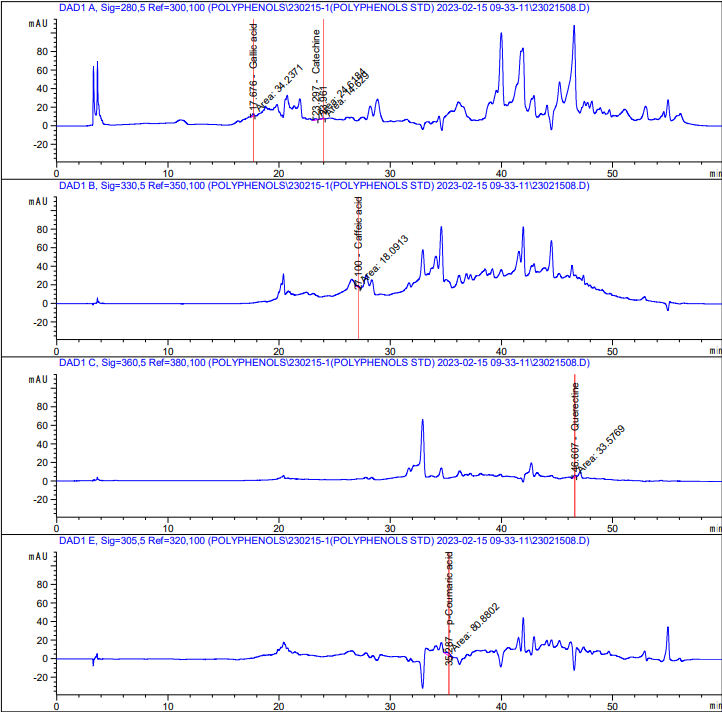


**(B)**


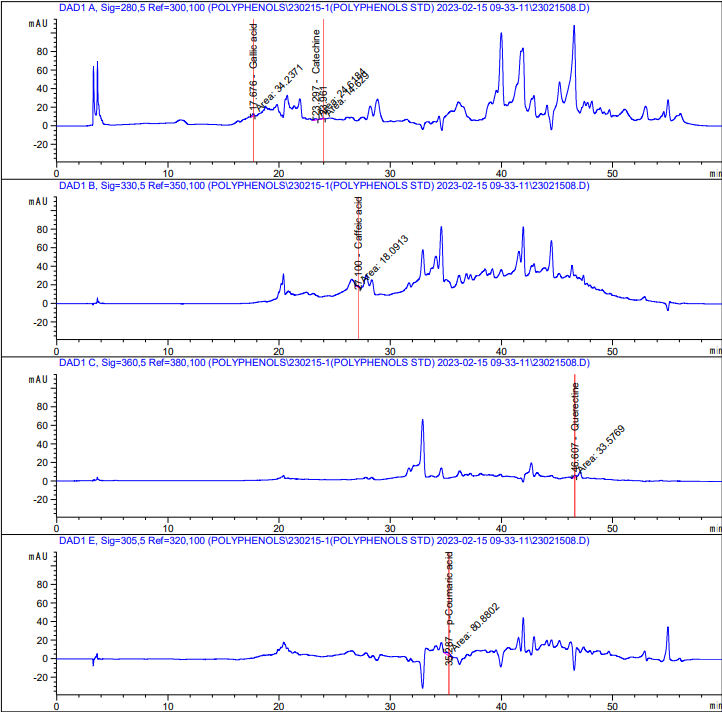


Figure S2: Representative chromatogram of the HPLC analysis performed on Sesame Seed Coat (SSC) extract: **(A)** first run and **(B)** second run.


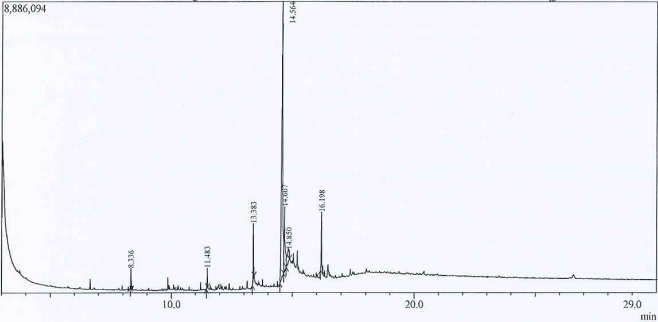


Figure S3. GC-MS chromatogram of Sesame Seed Coat (SSC).

Table S1. Effects of Sesame Seed Coat (SSC) on *L. monocytogenes* and *E. coli* O157:H7 in milk stored at 4°C and 10°C.

| **Day** | **Temperature**  **(**℃**)** | ***L. monocytogenes*** | | ***E. coli* O157:H7** | |
| --- | --- | --- | --- | --- | --- |
|  |  | **Control** | **SSC Extract** | **Control** | **SSC Extract** |
| 0 | 4 | 4.52 ± 0.02 | 4.52 ± 0.02 | 4.06 ± 0.06 | 4.06 ± 0.06 |
|  | 10 | 4.68 ± 0.10 | 4.68 ± 0.10 | 3.79 ± 0.12 | 3.79 ± 0.12 |
| ***p-value*** | | ***0.255*** | ***0.255*** | ***0.109*** | ***0.109*** |
| 1 | 4 | 5.53 ± 0.08 | 4.29 ± 0.15 | 3.41 ± 0.06 | 3.46 ± 0.24 |
|  | 10 | 5.85 ± 0.10 | 4.81 ± 0.02 | 4.32 ± 0.12 | 3.58 ± 0.16 |
| ***p-value*** | | ***0.06*** | ***0.067*** | ***0.002***** | ***0.687*** |
| 3 | 4 | 7.34 ± 0.02 | 4.00 ± 0.00 | 5.69 ± 0.03 | 4.58 ± 0.16 |
|  | 10 | 7.11 ± 0.05 | 3.93 ± 0.07 | 6.89 ± 0.39 | 4.73 ± 0.09 |
| ***p-value*** | | ***0.016**** | ***0.423*** | ***0.091*** | ***0.479*** |
| 7 | 4 | 7.96 ± 0.08 | 3.74 ± 0.14 | 7.87 ± 0.00 | 4.61 ± 0.14 |
|  | 10 | 8.24 ± 0.07 | 3.79 ± 0.26 | 8.37 ± 0.06 | 7.47 ± 0.09 |
| ***p-value*** | | ***0.057*** | ***0.855*** | ***0.001****** | ***<0.001****** |

The *p*-values were calculated such that *p* >0.05 was not significant, **p* <0.05, ***p* <0.01, and ****p* <0.001, where *, **, and *** denote levels of significance difference.

Supplementary Table S2. Effects of NE and NE-SSC on *L. monocytogenes* and *E. coli* O157:H7 in milk stored at 4°C and 10°C.

| **Day** | **Temperature** | ***Bacteria*** | **NE** | | | | **NE-SSC** | | | |
| --- | --- | --- | --- | --- | --- | --- | --- | --- | --- | --- |
|  |  |  | **0%** | **3%** | **7%** | **10%** | **0%** | **3%** | **7%** | **10%** |
| 0 | 4℃ | *L. monocytogenes* | 4.41 ± 0.06 | 4.41 ± 0.06 | 4.41 ± 0.06 | 4.41 ± 0.06 | 4.41 ± 0.06 | 4.41 ± 0.06 | 4.41 ± 0.06 | 4.41 ± 0.06 |
|  |  | *E. coli O157:H7* | 3.99 ± 0.02 | 3.99 ± 0.02 | 3.99 ± 0.02 | 3.99 ± 0.02 | 3.99 ± 0.02 | 3.99 ± 0.02 | 3.99 ± 0.02 | 3.99 ± 0.02 |
|  |  | ***p-value*** | ***0.002***** | ***0.002***** | ***0.002***** | ***0.002***** | ***0.002***** | ***0.002***** | ***0.002***** | ***0.002***** |
| 1 |  | *L. monocytogenes* | 5.50 ± 0.06 | 5.48 ± 0.04 | 5.16 ± 0.08 | 4.92 ± 0.04 | 5.50 ± 0.06 | 5.39 ± 0.02 | 5.03 ± 0.16 | 4.63 ± 0.02 |
|  |  | *E. coli O157:H7* | 4.37 ± 0.05 | 4.44 ± 0.11 | 4.15 ± 0.16 | 4.10 ± 0.06 | 4.37 ± 0.05 | 4.34 ± 0.11 | 4.15 ± 0.17 | 3.89 ± 0.22 |
|  |  | ***p-value*** | ***<0.001****** | ***<0.001****** | ***0.005***** | ***<0.001****** | ***<0.001****** | ***0.009***** | ***0.021**** | ***0.028**** |
| 3 |  | *L. monocytogenes* | 7.53 ± 0.05 | 7.38 ± 0.07 | 6.51 ± 0.03 | 5.45 ± 0.08 | 7.53 ± 0.05 | 7.02 ± 0.06 | 5.59 ± 0.02 | 5.00 ± 0.03 |
|  |  | *E. coli O157:H7* | 6.02 ± 0.04 | 5.69 ± 0.11 | 4.87 ± 0.08 | 4.48 ± 0.10 | 6.02 ± 0.04 | 5.60 ± 0.00 | 4.76 ± 0.03 | 4.14 ± 0.04 |
|  |  | ***p-value*** | ***<0.001****** | ***<0.001****** | ***<0.001****** | ***0.002***** | ***<0.001****** | ***0.002***** | ***<0.001****** | ***<0.001****** |
| 7 |  | *L. monocytogenes* | 8.09 ± 0.04 | 8.06 ± 0.03 | 7.52 ± 0.18 | 6.88 ± 0.10 | 8.09 ± 0.04 | 8.13 ± 0.06 | 7.14 ± 0.10 | 5.46 ± 0.09 |
|  |  | *E. coli O157:H7* | 7.66 ± 0.03 | 8.09 ± 0.21 | 5.47 ± 0.54 | 4.25 ± 0.01 | 7.66 ± 0.03 | 7.58 ± 0.04 | 5.24 ± 0.26 | 3.72 ± 0.07 |
|  |  | ***p-value*** | ***<0.001****** | ***0.915*** | ***0.023**** | ***<0.001****** | ***0.0477**** | ***0.002***** | ***0.002***** | ***<0.001****** |
| 0 | 10℃ | *L. monocytogenes* | 4.41 ± 0.06 | 4.41 ± 0.06 | 4.41 ± 0.06 | 4.41 ± 0.06 | 4.41 ± 0.06 | 4.41 ± 0.06 | 4.41 ± 0.06 | 4.41 ± 0.06 |
|  |  | *E. coli O157:H7* | 3.99 ± 0.02 | 3.99 ± 0.02 | 3.99 ± 0.02 | 3.99 ± 0.02 | 3.99 ± 0.02 | 3.99 ± 0.02 | 3.99 ± 0.02 | 3.99 ± 0.02 |
|  |  | ***p-value*** | ***0.002***** | ***0.002***** | ***0.002***** | ***0.002***** | ***0.002***** | ***0.002***** | ***0.002***** | ***0.002***** |
| 1 |  | *L. monocytogenes* | 6.07 ± 0.11 | 5.58 ± 0.07 | 5.30 ± 0.02 | 5.04 ± 0.03 | 6.07 ± 0.11 | 5.26 ± 0.05 | 4.68 ± 0.01 | 3.99 ± 0.05 |
|  |  | *E. coli O157:H7* | 5.11 ± 0.06 | 4.73 ± 0.04 | 4.33 ± 0.03 | 4.12 ± 0.07 | 5.11 ± 0.06 | 3.92 ± 0.26 | 3.57 ± 0.16 | 4.58 ± 0.05 |
|  |  | ***p-value*** | ***0.001****** | ***<0.001****** | ***<0.001****** | ***<0.001****** | ***<0.001****** | ***0.007***** | ***0.002***** | ***<0.001****** |
| 3 |  | *L. monocytogenes* | 7.79 ± 0.12 | 7.33 ± 0.12 | 6.43 ± 0.08 | 5.90 ± 0.20 | 7.79 ± 0.12 | 7.12 ± 0.08 | 5.56 ± 0.14 | 4.27 ± 0.05 |
|  |  | *E. coli O157:H7* | 7.05 ± 0.06 | 6.95 ± 0.03 | 5.85 ± 0.00 | 4.46 ± 0.03 | 7.05 ± 0.06 | 6.67 ± 0.05 | 4.87 ± 0.03 | 5.11 ± 0.05 |
|  |  | ***p-value*** | ***0.006***** | ***0.034**** | ***0.002***** | ***0.017**** | ***0.006***** | ***0.011**** | ***0.008***** | ***<0.001****** |
| 7 |  | *L. monocytogenes* | 8.04 ± 0.02 | 8.24 ± 0.05 | 7.92 ± 0.04 | 7.46 ± 0.03 | 8.04 ± 0.02 | 8.21 ± 0.11 | 7.61 ± 0.07 | 6.23 ± 0.05 |
|  |  | *E. coli O157:H7* | 8.87 ± 0.02 | 8.54 ± 0.11 | 8.01 ± 0.04 | 5.11 ± 0.06 | 8.87 ± 0.02 | 8.23 ± 0.01 | 5.20 ± 0.10 | 4.14 ± 0.07 |
|  |  | ***p-value*** | ***<0.001****** | ***0.075*** | ***0.191*** | ***<0.001****** | ***<0.001****** | ***0.896*** | ***<0.001****** | ***<0.001****** |

The *p*-values were calculated such that *p* >0.05 was not significant, **p* <0.05, ***p* <0.01, and ****p* <0.001, where *, **, and *** denote levels of significance difference.
